# Open-source Web Portal for Managing Self-reported Data and Real-world Data Donation in Diabetes Research: Platform Feasibility Study

**DOI:** 10.2196/33213

**Published:** 2022-03-31

**Authors:** Drew Cooper, Tebbe Ubben, Christine Knoll, Hanne Ballhausen, Shane O'Donnell, Katarina Braune, Dana Lewis

**Affiliations:** 1 Department of Pediatric Endocrinology and Diabetes Charité – Universitätsmedizin Berlin Berlin Germany; 2 Berlin Institute of Health Berlin Germany; 3 Dedoc Labs GmbH Berlin Germany; 4 School of Sociology University College Dublin Dublin Ireland; 5 Institute of Medical Informatics Charité – Universitätsmedizin Berlin Berlin Germany; 6 OpenAPS Seattle, WA United States

**Keywords:** diabetes, type 1 diabetes, automated insulin delivery, diabetes technology, open-source, patient-reported outcomes, real-world data, research methods, mixed methods, insulin, digital health, web portal

## Abstract

**Background:**

People with diabetes and their support networks have developed open-source automated insulin delivery systems to help manage their diabetes therapy, as well as to improve their quality of life and glycemic outcomes. Under the hashtag *#WeAreNotWaiting*, a wealth of knowledge and real-world data have been generated by users of these systems but have been left largely untapped by research; opportunities for such multimodal studies remain open.

**Objective:**

We aimed to evaluate the feasibility of several aspects of open-source automated insulin delivery systems including challenges related to data management and security across multiple disparate web-based platforms and challenges related to implementing follow-up studies.

**Methods:**

We developed a mixed methods study to collect questionnaire responses and anonymized diabetes data donated by participants—which included adults and children with diabetes and their partners or caregivers recruited through multiple diabetes online communities. We managed both front-end participant interactions and back-end data management with our web portal (called *the Gateway*). Participant questionnaire data from electronic data capture (REDCap) and personal device data aggregation (Open Humans) platforms were pseudonymously and securely linked and stored within a custom-built database that used both open-source and commercial software. Participants were later given the option to include their health care providers in the study to validate their questionnaire responses; the database architecture was designed specifically with this kind of extensibility in mind.

**Results:**

Of 1052 visitors to the study landing page, 930 participated and completed at least one questionnaire. After the implementation of health care professional validation of self-reported clinical outcomes to the study, an additional 164 individuals visited the landing page, with 142 completing at least one questionnaire. Of the optional study elements, 7 participant–health care professional dyads participated in the survey, and 97 participants who completed the survey donated their anonymized medical device data.

**Conclusions:**

The platform was accessible to participants while maintaining compliance with data regulations. The Gateway formalized a system of automated data matching between multiple data sets, which was a major benefit to researchers. Scalability of the platform was demonstrated with the later addition of self-reported data validation. This study demonstrated the feasibility of custom software solutions in addressing complex study designs. The Gateway portal code has been made available open-source and can be leveraged by other research groups.

## Introduction

Under the hashtag *#WeAreNotWaiting*, people with diabetes and their families have come together to develop and support the use of open-source automated insulin delivery systems (also called do-it-yourself artificial pancreas systems). With insulin pumps and data from continuous glucose monitoring, automated insulin delivery systems are able to automate insulin dosing in response to glucose levels through algorithmic prediction [1-4]. With an estimated >10,000 individuals using open-source automated insulin delivery worldwide, there is a wealth of data produced from these systems in real-world settings [5].

Web-based data repositories, such as Nightscout, allow users to collect, upload, review, analyze, and share data from open-source automated insulin delivery systems with their caregivers and health care teams [6]. Until recently, data uploaded to these sites were rarely used for research, which left an important source of real-world evidence largely untapped. Open-data platforms, such as Open Humans [7], allow users to anonymously donate their data from repository sites for use in research [7-9]. Data from Open Humans have previously been used in research and increasingly to evaluate open-source automated insulin delivery [8].

An international consortium of patient innovators, clinicians, social scientists, computer scientists, and patient advocacy organizations initiated a project called OPEN (Outcomes of Patients’ Evidence with Novel, Do-it-Yourself Artificial Pancreas Technology [10,11])) and investigated the *#WeAreNotWaiting* movement and open-source automated insulin delivery use, which led to a web-based survey [12].

It is common practice to use tools such as REDCap for electronic data capture and management in the implementation of web-based surveys. However, it is not possible to achieve required flexibility and user friendliness using such tools alone. The overall aim of this study was to assess the feasibility of developing a platform that would enable participants to share anonymized retrospective diabetes data in addition to completing surveys.

## Methods

### Study Design

The study design and linkage of multiple elements—including follow-up and satellite projects—is complex. The study concept contained an analysis of real-world diabetes data, and a survey that included questionnaires that collected basic demographic data, self-reported clinical outcomes, and responses to open-ended questions, as well as assessments of quality of life (Pediatric Quality of Life Inventory, World Health Organization- Five Well-Being Index), depression and anxiety (Depression Anxiety Stress Scale), sleep quality (Pittsburgh Sleep Quality Index), problem areas in diabetes (Problem Areas in Diabetes scale), fear of hypoglycemia (Hypoglycemia Fear Survey-II), impact of diabetes (Diabetes Attitudes, Wishes, and Needs), diabetes treatment satisfaction (Diabetes Treatment Satisfaction Questionnaire), diabetes well-being, partner diabetes distress, hesitation around automated insulin delivery systems (DIWHYnot), and the effects of the COVID-19 pandemic on diabetes management and quality of life.

The study included participants who self-identified as an adult or adolescent with diabetes, and caregiver or partner of a person with diabetes. Furthermore, both users and nonusers of open-source automated insulin delivery were included. At a later stage in the study, adult participants were also provided the option to independently validate their self-reported health data and clinical outcomes by their health care professional (endocrinologist, pediatric endocrinologist, diabetes educator or specialist nurse). Thus, the study was made up of 3 major elements: a survey containing questionnaires alone, device data donation on Open Humans, and a linked follow-up study on health care professional–validated health data and clinical outcomes.

### Platform Requirements

The nature of this research—a real-world study with human participants—required that data management be compliant with European Union General Data Protection Regulations [13] and that risks related to data sharing for the individual be minimized (pseudonymization, deidentification, informed consent, and right to withdraw). Enabling participants to join follow-up studies without storing their personal information also necessitated a custom solution for pseudonymous data management. Safely and securely managing data from multiple data streams also presented a unique challenge.

Making study participation simple required the development of a web portal for users. Such a web portal needed to also act as a formalized system of automated data matching between multiple data sets. The first objective in creating the platform—the Gateway—was linking questionnaire responses in REDCap to optionally donated device data in Open Humans. The second was for this platform to link data from participants to their partners or health care professionals. The final objective was that the entire process be anonymized and General Data Protection Regulation–compliant.

### Front-end Architecture

**Figure 1:**
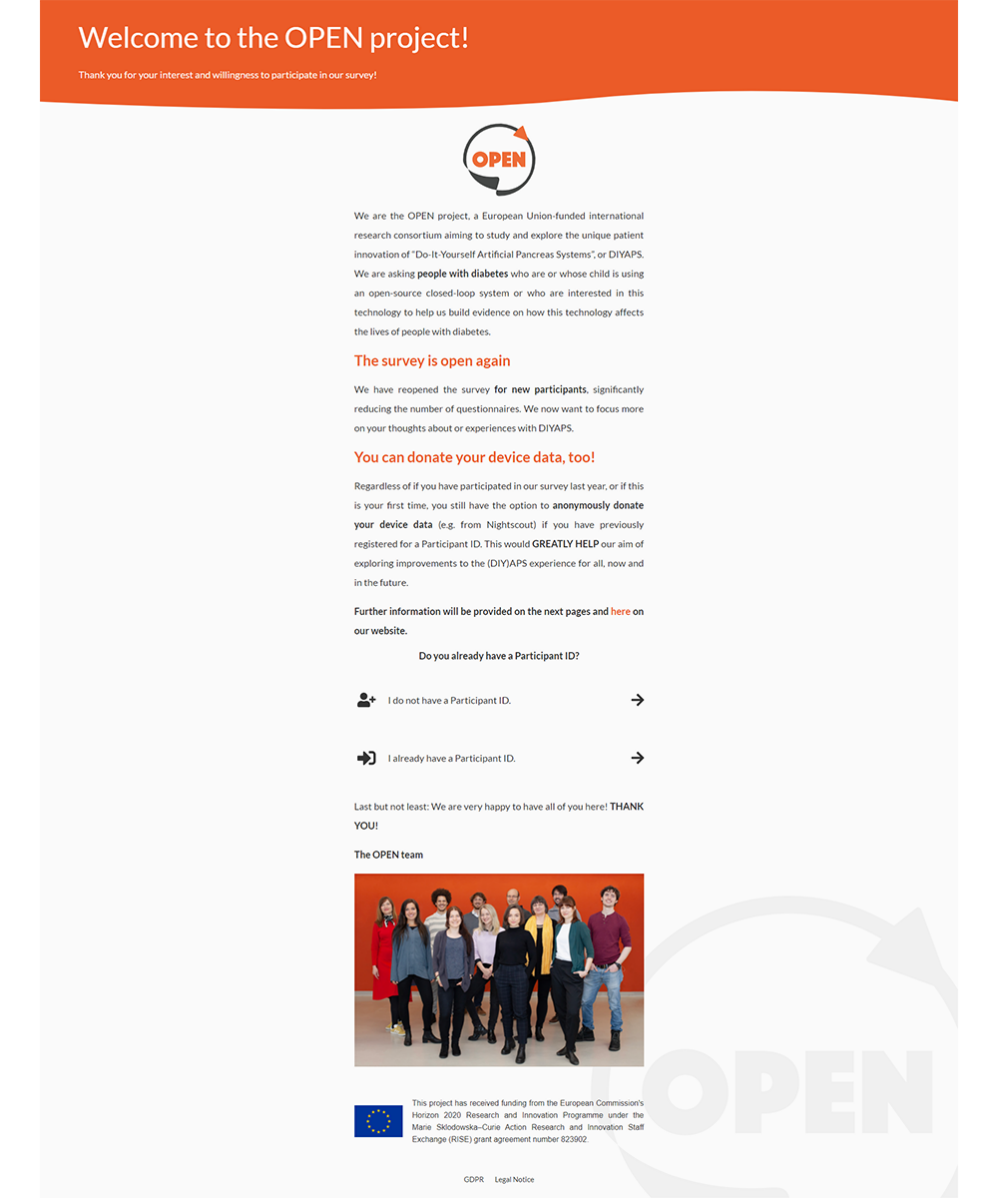
Landing page for the Outcomes of Patients’ Evidence with Novel, Do-it-Yourself Artificial Pancreas Technology (OPEN) project.

To users, the Gateway was a landing page (Figure 1) with a simple graphical user interface through which participants selected the profile with the appropriate characteristics (person with diabetes or caregiver of a person with diabetes; user or nonuser of automated insulin delivery) and were provided a unique *Participant ID*. Participants were informed of their rights regarding their survey data and optionally donated diabetes data and could then sign an electronic form if they wished to consent.

**Figure 2:**
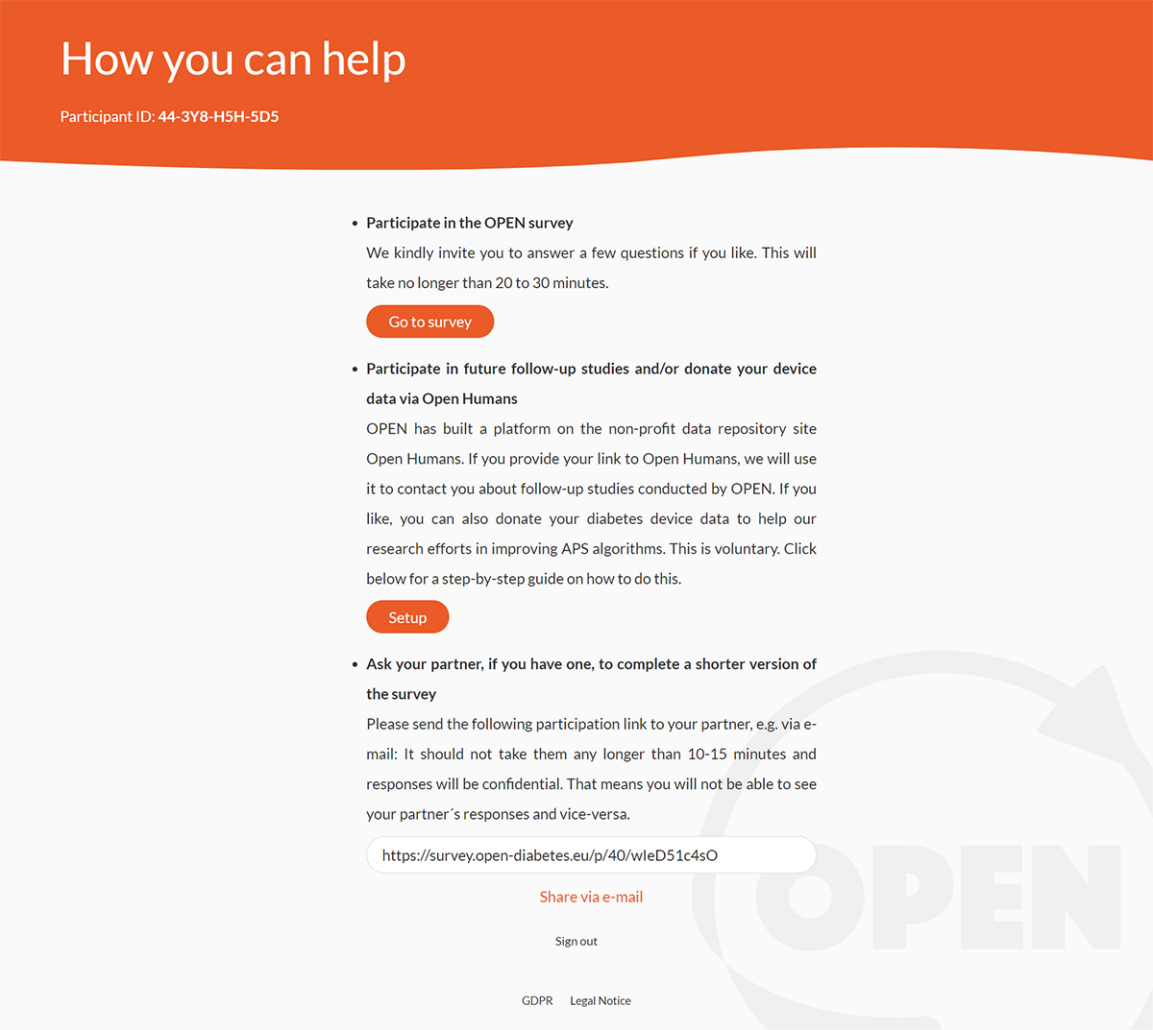
The Gateway webpage where participants can participate in the survey, donate their medical device data to Open Humans, or ask their partner to participate in the survey.

Participants responded to a sequence of questionnaires, and upon completion, they were asked if they wished to donate anonymized diabetes data and were provided with a survey link to send to other parties (eg, partners, parents, and health care providers) (Figure 2).

Responses to questionnaires were logged using REDCap (Vanderbilt University [14,15]). For privacy reasons, we did not use any device data cloud storage identifiers directly, as personal accounts may not be secure or anonymous. Rather, we managed medical device data donation through Open Humans with a specific project for OPEN [7]. OPEN positively evaluated the ability to communicate anonymously with study participants to notify them about follow-up studies, which is why Open Humans was chosen in addition to its ability to facilitate anonymized data donation. A *record ID* was generated for each participants’ survey response in REDCap, and an anonymous *Project Member ID* was generated when they joined the OPEN project on Open Humans. The Participant ID was used to link the record ID and Project Member ID within the Gateway.

### Back-end Architecture

The platform was developed using an open-source framework (Ktor, version 1.4.0; JetBrains [16]) in the Kotlin programming language. SQL data were translated (Exposed, version 0.26.1; JetBrains [17]) to Kotlin data types and stored using connection pooling (ie, opening as many database connections as necessary for reliable operation) (HikariCP, version 3.4.5; Brett Wooldridge [18]). Exposed and HikariCP support various databases by using the Java Database Connectivity interface [19], which added additional flexibility to the Gateway; for production, MariaDB [20] was chosen.

The database contained a table with the record ID and the Project Member ID for every survey participant. Application programming interfaces (APIs) were used to interact with these services to access survey and device data; data from these services were not stored in the database itself. In REDCap, each survey had an additional Gateway Instrument variable used to store each Participant ID as a backup measure, as well as additional survey information (eg, participant group, adult or caregiver, user or nonuser), which was used to establish branching logic sequences within specific surveys.

When a participant started the survey for the first time, REDCap’s *import record* API was initiated to create a new record containing that participant’s information (such as Participant ID and participant group). In that API call, the *Autogenerate record ID* flag was enabled, so that a new record was created instead of an existing record being edited, and the new record ID was returned in the API response. The record ID was then stored in the database; multiple record IDs could be stored for a single Participant ID, allowing for implementation of multiple surveys and follow-up studies. To send the user to the survey, another API call was made to REDCap to export the survey queue link for that given record ID and redirect the user.

### Participant ID

The Participant ID was formatted as *1-222-222-222*, where the first number was a consecutive counter (eg, first generated ID: 1, second ID: 2, 100th ID: 100), followed by a 9-digit secret number. The counter was generated by the SQL *auto-increment* feature, and the secret number was randomly generated using a random number generator.

A 9-digit secret number was included to minimize the risk that an unauthorized person could inadvertently or intentionally compromise survey data. For security reasons, the Gateway did not provide any information (questionnaire responses or device data) except for auxiliary status messages (eg, whether the survey has been completed or not), so that no confidential or personal data were exposed in the event that Participant IDs were accidentally made public.

Participants were advised to securely record their Participant ID, because this number allowed participants to start, stop, and resume the survey at any time, and link to the OPEN project on Open Humans.

### Authorization

An authorization protocol (OAuth [21]) created for third-party apps to access APIs without requiring app passwords from users—thus creating secure authorization flows—allowed access to and between the Gateway, REDCap, and Open Humans.

The authorization flow was implemented using Ktor’s built-in OAuth tool (OAuth, JetBrains [22]). When participants completed the survey, they were invited to donate their diabetes data to the OPEN project on Open Humans. To initiate this process, OAuth first referred participants to a URL on Open Humans where they can register or sign in to Open Humans and join the OPEN project, thereby granting the Gateway access to their data. After this step, the user was redirected back to the Gateway, with a *bearer token* in the URL. The Gateway recognized the token and traded it in at Open Humans for an access token and a refresh token. The access token was used to access the user's data—the refresh token provided a new access token (and refresh token) once the current access token expired. These tokens were stored in the Gateway’s database.

### Data Set Linkage

Linkage between REDCap records and Open Humans data sets was accomplished by storing the survey record ID and the Project Member ID in the same row as the Participant ID (Table 1) or with a reference using a foreign key. In SQL, every table has a column with a primary key whose values must be unique, which therefore allow a specific row to be referenced without conflict. This is usually just a counter (the first part of the Participant ID), which allows an entry to be referenced from another table. The foreign key is a special constraint that ensures the entry with a given ID exists and that can automatically delete and update an entry if its reference is altered.

**Table 1:** Data structure of a table of the Gateway database. (Data in the table are an example and not from study participants.)

Consecutive counter, *id*	9-digit secret	Participant group (0–6)^a^, *enrollment_type*	REDCap Record ID, *survey_record_id*	Open Humans Project Member ID, *project_member_id*	Access token, *access_token*	Refresh token, *refresh_token*	Unix timestamp (milliseconds), *expires_at*
1	5DBJ4D9R7	2	2	NULL^b^	NULL	NULL	NULL
2	G253LY4VC	1	1	79565297	YmtpPHHCug8FgVkQBvmszyP4nmXu6c	ZPhUY2pK85vvYuvhTr8qbEAtaCGAks	1606799777655
3	290FA1D9B	0	3	NULL	NULL	NULL	NULL

^a^0 indicates an adult using open-source automated insulin delivery,1 a nonuser adult, 2 a parent of a child user, 3 a parent of a child nonuser, 4 a teenage user, 5 a partner of an adult user, and 6 a partner of an adult nonuser.

^b^NULL indicates that there are no entry data.

### Hosting

The Gateway is hosted on a virtual storage server, running CentOS [23] and Docker [24]. The Docker image for the Gateway was created based on the official OpenJDK [25] image published on Docker Hub by including the compiled Gateway executable file and the MariaDB Java Database Connectivity [19] connector, whereas the official MariaDB image was used unmodified. A volume to store the database files was created, and both containers were connected using a bridge network. The Gateway container exposed the default ports 80 and 443 for HTTP to be accessed publicly by the participants. TLS (Transport Layer Security) certificates were retrieved from Let’s Encrypt—a nonprofit certificate authority—using Certbot, which proved domain ownership using the ACME (Automatic Certificate Management Environment) protocol, and were mounted into the container [26,27].

### Participant Recruitment

A group of 18 people with, or caregivers and partners of people with, diabetes were recruited to pilot test the platform prior to survey launch. Their responses and data were not included in the final data set.

For the final data set, we sought adults (aged ≥18 years) with diabetes (type 1, 2, or other), caregivers of children and adolescents (aged 3-17 years) with diabetes, and partners or health care professionals of people with diabetes. Participants were recruited via multiple online communities for diabetes, including Facebook groups (such as multinational Looped groups, AndroidAPS users, CGM in the cloud, Nightscout Deutschland), and through the OPEN project website, social media accounts, and Diabetes Daily.

### Participant Roles

Upon survey completion, participants were able to send survey links to their partners or caregivers, inviting them to participate in the study. Survey responses from partners or caregivers were linked via the Participant ID to the original participant; partners were linked to adults with diabetes, and caregivers were linked to adolescents with diabetes.

Health care professionals were added at a later stage (while the study was still ongoing). Health care professionals could be invited by people with diabetes to validate their self-reported data by providing information on comorbidities, most recent hemoglobin A_1c_ level, and episodes of severe hypoglycemia and diabetic ketoacidosis based on clinical records. Participants were asked to provide consent for the release of these data by their health care professionals by signing a physical consent form that was given to health care professionals directly and stored in participant health records.

### Ethical Approval and Data Privacy

Survey and data donation components of the study were approved by the Life Sciences Human Research Ethics Committee at University College Dublin (LS-20-37).

These study elements are in compliance with data regulation standards of the European Union General Data Protection Regulation. Open Humans is in compliance with regional data privacy laws, particularly those of the United States and European Union. Prior to participation in the study, participants electronically signed an agreement stating that their authorization of data sharing may waive their countries’ data privacy laws.

## Results

**Figure 3:**
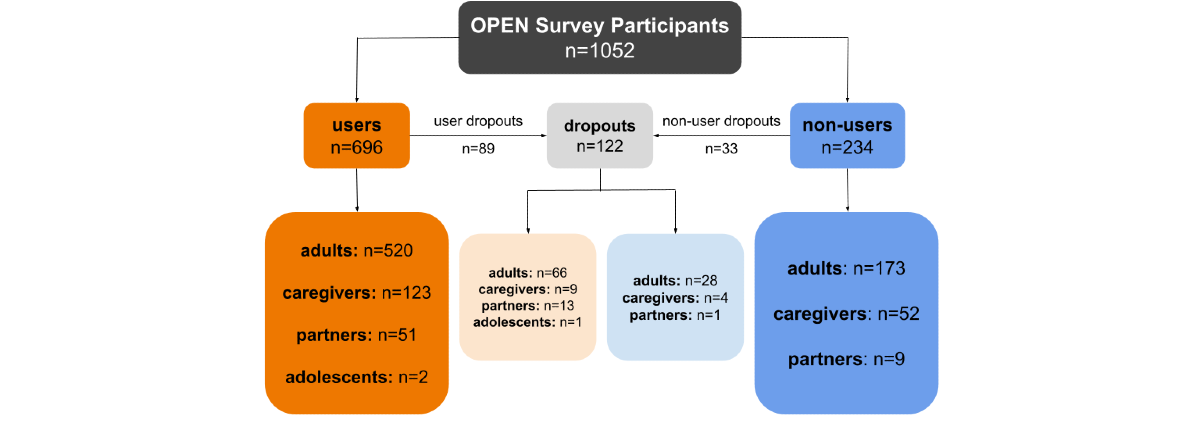
Flow diagram of study participation, prior to the addition of health care professional validation. OPEN: Outcomes of Patients’ Evidence with Novel, Do-it-Yourself Artificial Pancreas Technology.

By the survey’s close at the end of November 2020, a total of 1052 unique individuals had accessed the Gateway (Figure 3; Table 2), of whom 930 completed at least one questionnaire (users: 696/930, 74.8%; nonusers: 234/930, 25.2%).

**Table 2:** Participants who completed at least one questionnaire prior to addition of the health care professional validation element.

Participant type	Users (n=696), n (%)	Nonusers (n=234), n (%)	All (n=930), n (%)
Adults	520 (55.9)	173 (18.6)	693 (74.5)
Adolescents	2 (0.2)	0 (0.0)	2 (0.2)
Caregivers	123 (13.2)	52 (5.6)	175 (18.8)
Partners	51 (5.5)	9 (1.0)	60 (6.5)

**Figure 4:**
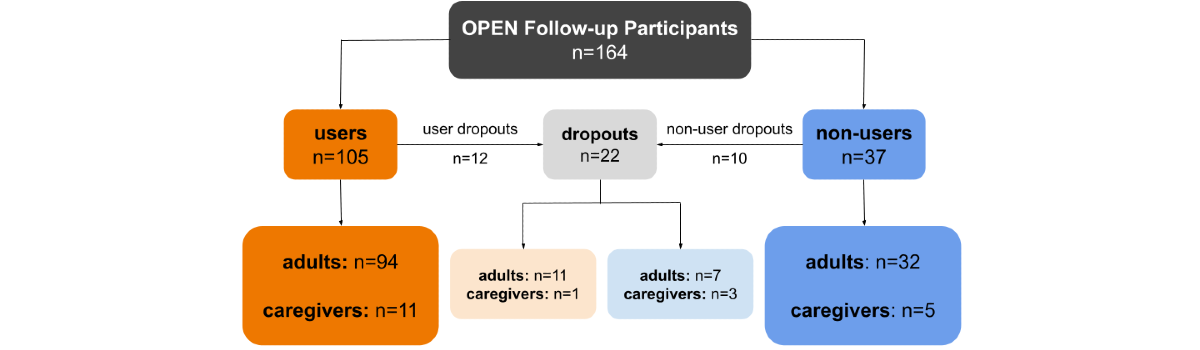
Flow diagram of study participation, with the addition of health care professional validation. OPEN: Outcomes of Patients’ Evidence with Novel, Do-it-Yourself Artificial Pancreas Technology.

After the Gateway was extended to enable health care professional validation of self-reported clinical outcomes, an additional 164 individuals visited the Gateway page, of whom 20 did not proceed to the survey and 2 dropped out during the first questionnaire; therefore, 142 participants (users: 105/142, 73.9%; nonusers: 37/142, 26.1%) completed at least one questionnaire (Figure 4; Table 3). A total of 7 participants allowed their health care professional to validate their clinical data—5 completed the survey before and 2 completed the survey after health care professional validation was added.

**Table 3:** Participants who completed at least one questionnaire after the addition of the health care professional validation element.

Participant type	Users (n=105), n (%)	Nonusers (n=37), n (%)	Total (n=142), n (%)
Adults	94 (66.2)	32 (22.5)	126 (88.7)
Caregivers	11 (7.7)	5 (3.5)	16 (11.3)

During the survey period, 137 individuals joined Open Humans. Of those 137 individuals, 97 participated in the survey, uploaded device data, and authorized the OPEN project to access their data on Open Humans; these 97 participants are represented within the larger group of 930 participants who completed at least one survey questionnaire. Open-source automated insulin delivery systems are highly individualized, allowing for a variety of pumps and continuous glucose monitoring systems to be used. Thus, data contained records from multiple different devices, including continuous glucose monitoring data from Dexcom (models G4, G5, or G6), Eversense, Medtronic (Guardian or Enlite models) and Freestyle Libre (model 2), as well as information about insulin delivery provided by pumps—Accu-Chek (Insight or Combo models), older Medtronic pumps, SOOIL Dana Diabecare (R or RS models), and Omnipod (Eros model). Continuous glucose monitoring data included timestamp entries of blood glucose levels, whereas pump data included information about insulin delivery such as extended boluses and temporary basal rates. Nonusers of open-source automated insulin delivery uploaded continuous glucose monitoring and pump data but did not have algorithmic automated insulin delivery data to donate. Individualized profiles from automated insulin delivery systems captured variable and algorithm output data, including changes to blood glucose targets, dosing decisions, carbohydrate entries, and general manual inputs.

## Discussion

### Principal Results

The Gateway fulfilled 3 main requirements to facilitate anonymous participation in multiple questionnaires and paired diabetes data donation: linking survey records in REDCap to Open Humans Project Member IDs as an optional extension, linking records from partners and health care professionals in addition to open-source automated insulin delivery users and nonusers, and making the entire process anonymized and General Data Protection Regulation–compliant.

Linking, the low cost of services, and familiarity were all related to the central objective of developing a platform for sharing anonymized diabetes data and completing surveys. Linking services improved ease of use for participants; open-source software is free and easier to expand upon (open repositories, direct communication with developers); and familiarity with the services (within research domains) provided a larger body of knowledge to pull from in experimental design, best practices for implementation, and data security. This last element is important—data privacy and security are critical when working with medical data for the protection of participants.

The initial approach was to let participants create an Open Humans account and join the OPEN project (thus generating a Project Member ID), then manually enter their Project Member ID into REDCap and create an identifier on their own with which their partner and health care professional could also join the survey. However, the Project Member ID from Open Humans could not be entered after the REDCap survey was completed, which made setting up data donation on Open Humans before starting the survey necessary. Furthermore, because registering for Open Humans, uploading data, and joining the OPEN project was a multistep process, participants could become fatigued and leave the study before reaching the questionnaires. There was additional concern that participants might accidentally reveal identifying information by creating linking identifiers, hence this approach was abandoned.

Another approach that we considered was requiring that all participants sign up for a personal account on Open Humans, to ensure that every participant had a Project Member ID available when beginning the survey. To minimize the burden of participation, we did not impose this requirement (ie, mandatory registration on a third-party platform), which could have limited the number of potential survey participants.

However, the use of Open Humans as a device data donation platform provided improved security and anonymity. We decided against using Nightscout accounts—or identifiers of any other device data cloud storage—for privacy reasons. Personal accounts may not be secure or anonymous; whereas, registration through Open Humans provided each participant with a unique anonymous ID and allowed for a standardized process of providing data to the OPEN project.

Existing tools and platforms were used; REDCap and Open Humans are both trusted, well-established, and have proven reliability, which has been demonstrated in previous studies [28-31]. Developing the Gateway was thus a feasible task as it only had to establish a linkage between data sets, whereas implementing questionnaires and data donation were predefined processes in their respective web-based services. Such a design kept overhead costs low relative to development and made use of familiar digital systems.

Completion of an electronic consent form was a prerequisite for participating in the study. While such a consent form was suitable for the bulk of the study—direct participant signatures were not required, only anonymous agreement to the study terms—the release of health care professionals from confidentiality (if participants participated in that component of the study) required a direct signature from the participant. An e-signature stored in the Gateway would have directly tied identifying information to participants’ survey responses and medical device data, compromising anonymity.

The decision was made to use physically signed consent forms that were given directly to health care professionals and ultimately stored with participants’ health records. These consent records were not available to OPEN—this enabled health care professionals to provide participant information without violating data protection regulations.

With the level of centralization afforded by the Gateway, it was feasible to add health care professional validation at a later stage of the study. It was only necessary to add another record ID from REDCap to the database and link it to the correct Participant ID; REDCap did not directly provide mechanisms for establishing such links; therefore, this would not have been possible without the Gateway.

Data were immediately accessible to the OPEN team at the end of data collection, with conditional access through an internal application process. Questionnaire responses were logged in REDCap and could be downloaded directly; similarly, Open Humans data could be downloaded directly from the OPEN project’s profile on Open Humans. The Gateway database—containing all participant IDs, survey record IDs, and Project Member IDs—was shared with OPEN members through a shared cloud drive. The Gateway was designed for adaptation to future studies and remains operational; the late addition of health care professional–validation demonstrated the functionality of linking new elements, allowing for continuous extensibility of the portal.

### Limitations

Despite the overall success of the study, there were some drawbacks to the final structure. To donate their diabetes data, participants first had to create Open Humans accounts, upload their data (which may involve first joining and utilizing an *uploader project*), and then join the OPEN Project on Open Humans (ie, authorize the OPEN project to access their device data). All steps had to be completed for the OPEN team to be able to access the anonymous donated diabetes data. The discrepancy between individuals who joined Open Humans and participants who completed the survey and authorized data donation could be attributed to all study elements being optional. Similar to the survey—where individuals across groups left before even completing the baseline demographic information (Figures 3 and 4)—individuals attempting to authorize the OPEN project to access their data may have exited the process before completion. Because all study elements were optional, individuals could choose to complete the survey but not authorize data access, authorize data access but not complete any questionnaires, complete both study elements, or exit before completing anything. The long list of questionnaires and multistep process of data authorization may have been too extensive for some individuals; this may have limited the potential amount of diabetes data captured.

While we thought that ensuring data privacy and anonymity could help to reduce the perceived burden of participation—based on the assumption that people would be more likely to provide detailed information if their identity remains private—there is evidence against this idea [32]. Additionally, the extensiveness of the study may have overpowered any potential reductions in perceived burden of participation due to anonymity; survey fatigue may have negated any retention achieved due to privacy. The presence of dropouts from each participant group is evidence that counters the argument that privacy precipitates participation.

In line with this, the potential risk of participants uploading simulated or falsified data was also considered. On one hand, anonymity theoretically makes tracing these participants more difficult. On the other hand, the number of steps required to produce authentic falsified data would be prohibitively complex. Most falsified automated insulin delivery data can be identified by researchers, as there are a number of elements (such as formatting, quantity and structure, algorithm decisions and variables) within data sets, which would create major barriers to generating authentic falsified data. To date, there are no reported issues of this occurring within research leveraging Open Humans. In general, it has been shown elsewhere [33-35] that real-world data are an important and robust source of information in addition to those from clinical trials. Furthermore, we screened both survey and device data for false entries and removed obvious outliers and erroneous entries where necessary.

While physical signatures were a feasible approach for obtaining consent from participants for their health care professionals to release medical data, the low number of participating health care professionals relative to survey participants may have been a consequence of adding a singular physical element to a study that is largely web-based. Participants may have been less willing to print out and personally send, rather than electronically sign, a form. Health care professional involvement was also the last element to be added to the study; this may have impacted participation. There are many potential factors resulting from the ongoing COVID-19 pandemic (maintaining safety precautions, continued changes to daily life, and carrying out vaccinations) that may have contributed to lower participation rates in the health care professional validation part of the study.

While not necessarily a limitation in this study, future studies may be impacted by tools and frameworks used by this study. Because of the developer’s familiarity with Ktor—which did allow for quick prototyping—any future developers working with this codebase that decide to replicate this approach may have to use a completely different toolchain that better fits their needs.

### Conclusion

The Gateway, as a portal made OPEN studies [10-12] both accessible for participants and manageable for researchers while maintaining General Data Protection Regulation compliance. Implementation of the disparate study elements was not necessarily complicated; creating the linkages between them required a creative solution, and scalability was also demonstrated with the later addition of health care professional validation of self-reported clinical outcomes. A practical mechanism for matching data sets and establishing links between disparate systems made this study and its extensions possible. In the future, custom software solutions such as the Gateway may become the norm in research with increasingly large data sets across disparate digital services.

## Abbreviations

API: application programming interface
OPEN: Outcomes of Patients’ Evidence with Novel, Do-it-Yourself Artificial Pancreas Technology

## Data Availability

After the completion of the OPEN project, requests to access study data can be made to SO. The Gateway codebase [36] is available for use by researchers aiming to implement similar multimodal study designs.
